# Supplementary material for: Are carbon emissions trading and green financial instruments synergistic? -Comprehensive quantitative research based on content analysis
Source: PLoS One. 2024 Mar 7;19(3):e0298601. doi: 10.1371/journal.pone.0298601 (PMC10919601; doi:10.1371/journal.pone.0298601)
Supplement: S1 Data — (ZIP) [file pone.0298601.s001.zip › Data/地级市Z_F工作报告.zip!/╔╜╬≈/╨├╓▌╩╨/└┤╫╘╣1⁄2╓┌║┼í╛┬φ┐╦╩2╛▌═°í┐_2012.docx]

各位代表：

　　现在，我代表市人民政府向大会报告政府工作，请予审议，并请市政协委员和其他列席会议人员提出意见。

　　一、2011年工作回顾

　　2011年是“十二五”开局之年。一年来，市政府在省委、省政府和市委的正确领导下，深入贯彻落实科学发展观，认真实施“3581”发展战略，着力推动转型跨越、赶队前行，全市经济社会发展和各项工作取得新的成绩。

　　全市生产总值完成554.5亿元，同比增长15%；全社会固定资产投资完成516亿元，同比增长35.1%；社会消费品零售总额完成185.5亿元，同比增长17.4%；财政总收入完成122.2亿元，同比增长35.5%；一般预算收入完成53.6亿元，同比增长26.5%；城镇居民人均可支配收入达到15924.6元，同比增长16.4%；农民人均纯收入达到4135元，同比增长20%。在完成经济社会发展目标任务的同时，市政府年初向全市人民承诺的实事全部落实兑现。

　　（一）项目建设实现新的突破。狠抓省市两级重点工程。年内完成投资491.6亿元，重点项目落地率301%，省级重点工程年度投资完成率117%。大力推进重大基础设施项目。五台山景区改造提升工程启动，云中河治理一期工程完工，忻保高速竣工通车，灵河高速全线开工，五台山飞机场建设前期工作基本完成。不断加大项目招商力度。中博会签约项目132个，签约资金2644亿元；农博会签约项目18个，协议引资34.4亿元；以岢岚县赴内蒙古招商为开端，以宁武县、繁峙县赴香港引资为标志，以县为单位“走出去”自主招商迈出新步伐。

　　（二）转型发展呈现强劲势头。加快发展8大产业。煤炭资源整合、煤矿兼并重组基本完成，原煤产量达到4625万吨，煤炭工业增加值完成110.5亿元；河曲电厂二期建成试运行，宁武、偏关、静乐、岢岚等县的7个风电项目开工建设，神池等3个风电项目并网发电；同德铝业100万吨氧化铝项目获批开工，金德成信铜钼矿项目加紧推进；忻州煤化工循环经济园区扩能升级，侨友化工顺酐项目取得新的进展，同德化工胶状乳化炸药项目完工达产，静乐“1830”项目开始试生产；蓝天煤粉锅炉扩建项目进展顺利，定襄法兰产业提升步伐加快；绿色农牧产品加工业得以壮大，新上农产品加工项目53个，纪元玉米公司成为国家级重点龙头企业，绿野牧业等12家企业入选省级重点龙头企业，年销售收入100万元以上的龙头企业达到143个；大力推进以五台山、雁门关、芦芽山等重点景区为依托的文化旅游产业发展，全市旅游总收入127.9亿元，同比增长20.4%；谋划建设了一批运煤专线、煤炭配送中心、煤炭集运站，煤炭物流业成为五寨等县新的增长点。全力实施60个重点转型技改项目，扎实推进重点园区建设。原平循环经济工业园区等5大园区初具规模，保德王家岭园区等5个园区加快规划。全面提升生态文明水平。淘汰19户企业160万吨落后产能，万元GDP能耗下降3.57%，完成考核目标；中心城市创建国家环保模范城工作全面启动，繁峙县城成功创建山西省环保模范城；忻州城区大气质量二级以上天数达到359天，其中一级天数157天，污染指数比上年下降1.39%；加大造林绿化力度，共完成营造林面积65.3万亩。

　　（三）“三农”工作迈出坚实步伐。认真贯彻落实各项强农惠农富农政策，市县财政农业奖补资金突破4亿元。全市粮食总产29.7亿斤，再创历史新高。加快发展现代农业。发展温室大棚2.58万亩，同比增长88%；发展经济林36万亩，同比增长80%；畜牧业总产值突破30亿元，同比增长10%；农产品加工销售收入达到37.3亿元，同比增长10%；新认证“三品一标”农产品147个，新发展“一村一品”专业村270个、农民专业合作社648个。加强农业基础设施建设。10座中小型水库除险加固工程完工，滹沱河灌区续建配套工程、忻定原小农水重点县建设、河保偏提黄灌溉工程顺利推进，“一村一井”工程全面启动，全市实灌面积达到171.68万亩。进一步改善农村面貌。建成新农村集中连片示范区14个，新发展新农村重点推进村234个。扎实推进扶贫开发。启动7个县的片区开发工程，加快实施138个整村推进项目，完成易地扶贫搬迁3.8万人，全年减贫7.51万人，全省易地扶贫搬迁工作现场会在我市召开。

　　（四）市域城镇化进程不断加快。加大中心城市建设力度。启动忻定原融入太原都市圈工作，初步完成《忻州市城市总体规划》修编，编制了《忻定原城市组群规划》；和平街改造工程如期竣工，市体育场大型人防工程奠基开工；第二热源厂二期工程建成投运，改造老旧供热管网30公里，城区天然气置换人工煤气超过1万户；成功申报国家可再生能源建筑应用示范城市。扎实推进城镇扩容提质行动。忻府区秀容古城系列改造一期工程完工，各县新区标准化建设和旧城综合整治步伐加快，15个省级重点镇建设工作全面铺开，繁峙县砂河镇、宁武县阳方口镇列入全省“百镇建设工程”示范镇。开工建设各类保障性住房13334套、89.55万平方米，超额完成年度任务。

　　（五）以综改为龙头的各项改革深入推进。编制完成《忻州市国家资源型经济转型综合配套改革试验两年行动方案》，原平市、宁武县分别列为省、市综改试点县，禹王煤化工循环经济园区、蓝天节能环保科技创新园区列为省综改试点园区。围绕产业转型、生态修复、城乡统筹、社会民生4大领域，统筹推进22个省市综改标杆项目。全力支持原平市、保德县进行扩权强县试点，县域经济发展的动力和活力进一步增强。积极稳妥推进事业单位改革，基本完成清理规范和科学分类任务。全面完成文化体制改革年度任务。推进医药卫生体制改革，完成五项医改年度任务。加快集体林权制度改革，林地产权明晰率达到99.74%。

　　（六）安全稳定形势持续明显好转。强化各级政府安全生产监管责任，认真执行省政府10大项12个行业118条安全生产规定，出台道路交通“双人包保、挂牌负责”等9项管理制度。高度重视煤矿安全生产，煤炭百万吨死亡率下降到0.06。深入开展隐患排查治理行动和安全生产百日大检查活动，对煤矿、尾矿库、危险化学品生产经营企业全部实行了挂牌责任制。扎实推进安全生产标准化建设，各类企业安全生产投入达到6.8亿元。全市安全生产形势稳定好转，事故死亡人数较上年减少18人，下降9.9%。深入开展“社会管理创新年”活动，健全完善社会治安防控体系，部署实施“平安忻州一号、二号”专项行动，社会治安形势保持稳定。深入开展信访积案化解活动，信访工作受到上级充分肯定。

　　（七）社会事业全面发展，人民生活进一步改善。大力发展教育事业。制订出台全市教育中长期发展规划纲要，全面完成义务教育标准化县建设任务，新建、改建和扩建标准化幼儿园34所，财政教育投入达到省定目标。加快发展卫生事业。市人民医院新址加快建设，市中医院医技康复大楼主体竣工，2所县级医院建成投运，10所县级医院主体竣工，实现了二甲医院“县县有”。积极发展文化体育事业。大力实施文化信息资源共享工程、乡镇文化综合服务工程、农村电影放映工程；成功举办第五届中国·忻州摔跤节。认真做好人口计生工作，考核指标全部达到省政府要求。进一步稳定和扩大就业。全年城镇新增就业3.51万人，失业人员再就业1.62万人，就业困难人员就业5012人，转移农村劳动力4.37万人，创业就业6800人，全部超过目标任务20%以上；城镇登记失业率3.3%，低于年度控制目标。全面加强社会保障。城镇居民社会养老保险试点县覆盖10个县（市），新型农村社会养老保险试点县覆盖10个县（市）；继续提高企业退休人员基本养老金，人均月增加227元；推进医疗保险市级统筹，进一步提高城镇职工、城镇居民医疗保险统筹基金支付比例，解决了国有关闭破产企业退休人员和特困企业职工参加基本医疗保险问题，减轻了群众就医负担；扩大失业保险金使用范围，参保人数增加到19.5万人；扩大工伤保险覆盖面，参保人数增加到14.8万人；提高城乡居民最低生活保障标准，城市低保居民人均每月增加25元，农村低保居民人均每月增加22元。农村新的“五个全覆盖”工程超额完成年度任务。为低收入农户每户免费供应1吨采暖煤落实到位。“家电下乡”和家电以旧换新受到人民群众的热切欢迎。

　　（八）民主法制和精神文明建设进一步加强。市政府认真执行市人大及其常委会的各项决议，主动接受市人大的工作监督、法律监督和市政协的民主监督，坚持和完善向人大报告工作制度和向政协通报工作制度，积极支持市政协参政议政。办理人大代表建议128件、政协委员提案265件，办结率均为100%。广泛征询民主党派、无党派代表人士以及群众团体和社团组织的意见，努力做到民主科学决策。启动“六五”普法工作。坚持依法行政，加强政府法制工作。改革行政审批制度，不断提高行政效能。认真落实政府系统党风廉政建设责任制，深入开展政风行风评议活动，切实加强行政监察和审计工作。煤焦领域反腐败专项斗争取得明显成效。全面贯彻民族宗教政策，依法管理民族宗教事务。深入开展国防教育，加强预备役建设和国防后备力量建设。军政军民团结不断巩固。我市第6次蝉联全国双拥模范城荣誉称号，岢岚县首次获得全国双拥模范县荣誉称号。完善基层民主制度，全面完成第九届村委会换届工作。精神文明创建活动成效显著，为经济社会发展提供了有力的思想文化保障。外事、侨务、人防、气象、地震、文物、档案工作得到加强，老龄、残疾人、红十字会等工作都取得了新的成效。

　　各位代表：回顾过去的一年，我们之所以能够较好地完成各项任务，是市委科学决策、正确领导的结果，是市人大、市政协积极支持、有力监督的结果，是各级人大代表和政协委员同心同德、群策群力的结果，是全市上下认真落实市委“3581”发展战略，团结奋斗、扎实工作的结果。在此，我代表市人民政府，向全市干部群众，向驻忻部队指战员、武警官兵和公安民警，向各民主党派、人民团体，向各级各类驻忻单位，向所有关心支持忻州发展进步的朋友们，表示衷心的感谢！

　　我们清醒地认识到，我市的发展面临更新思想观念与创优发展环境任务并重、强市与富民任务并重、转型与跨越任务并重、工业化与生态文明建设任务并重、城镇化与城乡统筹任务并重等艰巨的任务。我们要想尽快赶上全省发展水平，实现又好又快发展，就必须充分发挥我市的区位优势、资源优势、文化优势、后发优势，紧紧扭住转型跨越不动摇，以更大的勇气和胆略，坚持把解决发展不足的问题放在首位，加快做大经济总量；坚持把产业优化升级作为中心任务，努力走出资源型后发地区转型发展新路；坚持把县域发展作为重要抓手，努力实现全市城乡经济、社会、生态的全面协调持续进步；坚持把打造五台山名片作为龙头工程，充分发挥对文化旅游业的引领带动作用；坚持把扶贫开发作为重要而紧迫的任务，加快脱贫致富步伐，通过艰苦工作和不懈努力，在建设富裕文明、开放和谐、充满活力的新型工业旅游城市的征程上迈出新的更大的步伐，绝不辜负省委、省政府的厚望和全市人民的重托！

　　二、2012年工作安排

　　2012年是党的十八大胜利召开之年，是我市的“项目落地年”、“大干城建年”、“走出去年”和“基层组织建设年”，是转型跨越、赶队前行、充满希望、大有可为的一年。做好今年的政府工作，必须以中国特色社会主义理论体系为指导，深入贯彻落实科学发展观，认真实施“3581”发展战略，坚持科学发展的主题，紧扣加快转变经济发展方式的主线，把握稳中求进的工作总基调，以转型综改试验区建设为总抓手，全面推进以项目攻坚为重点的产业转型升级，以农民增收为重点的社会主义新农村建设，以特色旅游文化产业发展为重点的文化强市建设，以中心城市和大县城、示范镇建设为重点的城乡一体化发展，以保障和改善民生为重点的和谐社会建设，以干部作风转变为重点的政府自身建设，切实推动各项工作上台阶，以优异成绩迎接党的十八大胜利召开。

　　主要预期指标是：地区生产总值确保增长13%、力争达到15%左右，固定资产投资增长25%以上，社会消费品零售总额增长16%，进出口总额增长12%，财政总收入增长18%，一般预算收入增长16%，城镇居民人均可支配收入增长13%，农民人均纯收入增长14%，城镇新增就业岗位2.89万个，城镇登记失业率控制在4.2%以内，居民消费价格总水平涨幅控制在4%左右。这些指标是指导性的，可以根据形势变化适当调整。

　　约束性指标是：万元生产总值综合能耗下降3.5%，万元生产总值二氧化碳排放量下降3.7%，二氧化硫、化学需氧量、氨氮排放量分别下降2%、1.3%、1%，氮氧化物排放量实现零增长，烟尘、粉尘排放量下降3%，万元工业增加值用水量下降3.5%。这些约束性指标是硬任务、硬指标，必须完成。

　　实现上述目标，要在全面做好各项工作的同时，重点抓好以下工作：

　　（一）狠抓项目建设，为转型跨越发展提供有力支撑。

　　进一步扩大项目投资。今年安排省市两级重点工程213项，包括省重点工程42项、市重点工程171项，总投资概算2000亿元，年度计划完成553亿元，同比增长22.3%，占年度固定资产投资任务的85%以上。其中，产业项目157项，基础设施项目29项，民生项目27项。

　　强化基础设施建设。全面推进灵河高速公路、忻州环城高速公路、五台山飞机场、大西客运忻州段、五寨至河曲沙泉地方铁路、保德至兴县瓦塘地方铁路、静乐至娄烦静游地方铁路、108国道繁峙段一级路改造等重大基础设施项目建设，着力破解制约发展的瓶颈问题。

　　加快八大产业转型升级。煤炭产业要加大现代化矿井建设力度，在确保安全的前提下，原煤产量要达到5500万吨、力争突破6000万吨；启动重点煤矿“一矿一业”转型项目建设。电力产业要争取火电，提升水电，积极发展新能源发电，加快实施华能、大唐、国电等大集团在我市的风电项目，千方百计推进广宇2×350MW热电联产、定襄2×350MW热电联产、宁武2×300MW煤矸石电厂以及鲁能河曲电厂三期、同华轩岗电厂二期等重点项目。新材料产业要加快推进原平百万吨短流程铸造循环经济项目、忻府区10万吨煅烧高岭土扩建项目等18个重点项目。新型煤化工产业要加快推进静乐正联洁净改性型煤项目等12个重点项目。装备制造业要加快推进繁峙华茂100万吨特种钢升级改造项目等9个重点项目。绿色农牧产业要致力打造小杂粮和生态畜牧两大特色品牌，加快推进定襄龙门生态农业项目、代县金九州农产品项目、岢岚山地阳光沙棘全果素项目等31个重点项目。文化旅游产业要以五台山景区改造提升工程为龙头，改善基础设施，强化生态修复，规范市场秩序，优化旅游环境，确保把五台山改造提升工程建成全省转型综改试验区建设旅游领域的标杆项目；加快实施雁门关景区完善、芦芽山景区提标、沿黄景区开发、温泉旅游度假区改造各项重点工程，促进旅游与文化融合发展。现代物流业要充分发挥我市横跨东西、贯通南北的区位优势，加快建设运输平台、信息平台和储存配送平台，重点推进定襄商贸物流街区项目、宁武豪德汇通商贸物流城项目、五寨数字化配煤基地项目等20个重点项目。

　　加大项目攻坚力度。实行项目储备、招商、落地、开工、服务、考核“六位一体”，建立县（市、区）长联系“一园六企”制度。坚持开发区示范、园区带动、全民创业支撑，充分激发项目建设的活力。紧紧扭住项目攻坚，千方百计破解土地、环评、立项、资金难题。积极为企业生产经营搞好服务，竭力支持现有企业做大做强做优。通过借船出海、以企招商、以商招商，广泛吸引外地优秀企业到我市投资，真正使忻州成为“近者悦、远者来”的创业乐土和投资洼地，让项目的效益最大限度体现在本地。认真考核招商履约率、资金到位率和项目落地率，促进中博会签约项目、重点招商项目落地开工，尽快形成实物工作量和新的经济增长点。

　　（二）扎实做好“三农”工作，促进农民收入较快增长。

　　加快推进农业产业化。抓好产业基地、龙头企业、自主品牌3个关键，以工业化理念走出农业产业化路子。扭住生产、加工、营销3个环节，推进农业内部三次产业合理分工。加快发展优特杂粮产业，在环芦芽山、环五台山地区布局红芸豆、马铃薯、甜糯玉米、杂交谷4大特色杂粮基地，打造100平方公里小杂粮产业开发现代农业综合体。稳步发展设施蔬菜产业，重点支持有一定规模、能达产达效的园区，年内新增设施蔬菜1.3万亩。提升畜牧养殖产业水平，突出草食畜发展、养殖示范工程建设、畜产品安全3个重点，建设标准化养殖小区60个、规模养殖场100个，发展规模养殖户500户。大力实施“337”工程，积极培育壮大农产品加工龙头企业，力争农产品加工企业销售收入突破40亿元。大力发展“一村一品”，抓好农产品“三品一标”认证，打造具有忻州特色的自主品牌。加快农产品市场开发，重点建设10个农产品产地批发市场，发展100个购销组织和500个营销经纪人大户，把原平农副产品加工贸易园区建成全市第一个现代农业物流中心，把市农业信息中心网络建成全市第一个农产品大型电子商务平台，切实解决好农产品产销问题。加快推进农业科技创新，启动“一乡一站、一村一点”科技服务全覆盖工程，切实提高现代农业技术推广普及率。

　　加强农业基础设施建设。开工建设五寨清涟河、代县峨河河道治理工程，继续抓好滹沱河灌区节水项目和汾北灌区、孤山灌区节水改造工程，全面完成9座中小型病险水库除险加固工程，加快推进西部6县“一村一井”项目、小农水重点县建设工程、沿黄3县提黄灌溉工程，逐步破解水瓶颈问题。启动繁峙、代县、五台、定襄4县京津风沙源治理水保工程。大力提升农业物质技术装备水平，加快发展农机合作社和农机大户，进一步提高农业生产机械化程度。大力实施农业综合开发，提高土地生产能力。加大耕地开发整理力度，切实做到占补平衡。认真落实土地用途管理规定，确保耕地保有量不少于973万亩，确保基本农田不少于791.7万亩。

　　加大扶贫开发力度。坚持把产业发展作为扶贫攻坚的根本，把激活主体作为扶贫攻坚的关键，做到扶贫随着产业开发走、搬迁随着群众意愿走。认真贯彻落实中央和省委、省政府的要求，在抓好片区开发、教育扶贫、整村推进、产业扶贫工程以及集中连片地区扶贫开发试点工作的同时，坚定不移地把易地扶贫搬迁作为扶贫开发的治本之措来抓，年内完成易地扶贫搬迁3.2万人，实现搬得出、能致富、不反弹，确保全年减贫7万人。

　　提升新农村建设水平。科学制定、严格落实新农村建设规划，新建500个新农村重点推进村，集中打造10个“村民富、村庄美、村风好”的新农村连片建设示范区。大力发展农村公共事业，进一步提高农村教育、卫生、文化发展水平。高质量完成农村新的“五个全覆盖”工程任务。新解决10万农村居民的饮水安全问题。

　　不断激发农民主体活力。加强对农民的教育和引导，提高农民综合素质和自我发展能力。全年培训农民13万人，转移农村劳动力3.5万人，努力增加农民工资性收入。新发展农民专业合作社400个，不断提高农民的组织化程度。进一步优化农村金融生态环境，创新金融产品，搞好金融服务，为“三农”提供最大限度的金融支持。自觉坚持党对农村工作的领导，充分发挥村党支部的核心作用，不断完善村民自治制度，努力提高农村合作经济组织自我管理、自我发展的能力，切实激发农民自主发展的活力。

　　（三）认真落实“大干城建年”工作部署，加快市域城镇化步伐。

　　突出规划引领作用。坚持规划先行。认真执行忻州城市建设总体规划，抓紧制定忻州老城、云中新区的控制性详规，认真做好城市道路、供水、供热等专项规划。严把规划实施关，严审修建性规划，确保规划落到实处。

　　全力推进城区重点工程。开工建设“七路四桥”。力争傅山路、芦芽山路、云中山路北拓、城北街、北环街、云中河北路、云中河南路等7条道路8月份完工；力争芦芽山路桥、傅山路桥、云中路桥9月份完工，忻顿路桥完成设计。全面启动忻州市博物馆、图书馆、科技馆、艺术馆、体育馆、歌剧院等“五馆一院”工程，7月份开工建设。认真做好忻州城区创建省级园林城市工作，力争建成区新增绿化面积150万平方米，绿化覆盖率提高5个百分点。不断改善城区基础设施条件，进一步优化人居环境。

　　全面开展扩容提质大行动。抓好各县市新区建设，统筹推进旧城整治和城中村改造，切实提高水、气、热等基础设施保障水平。重点加快代县新城区、繁峙滨河新区、静乐河西区、保德西区建设步伐，扎实推进繁峙县砂河镇、宁武县阳方口镇两个“百镇建设工程”示范镇建设工作。启动各县市“三馆一院”建设。加快推进各县市户籍制度改革，放宽忻州城区落户条件，力争全市城镇化率提高1.5个百分点。切实提高城市管理水平，建立规范有序的城市建设工作秩序。

　　（四）加大节能减排力度，进一步改善生态环境。

　　进一步抓好减排治污。以减排、净空、净水、清洁为重点，大力实施绿色生态工程。加大工业源、农业源、生活源污染治理，严格控制重点区域和主要流域的污染排放。确保16座城镇生活污水处理厂正常运行，确保在建的7座垃圾处理场、城区垃圾焚烧发电项目建成投运，抓紧启动重点乡镇污水处理厂建设。加大机动车尾气治理力度，抓好采暖锅炉燃煤污染、建筑工地及道路施工扬尘污染控制工作。高度重视地下水和饮用水源保护，深化工业废水治理，全面加强滹沱河、峨河等重点流域水污染防治，确保消灭劣五类水体，全流域达到水环境功能要求，出境断面达到国家和省考核目标要求。大力开展城乡清洁行动，扎实推进农村环境连片整治，力争从根本上解决“垃圾围城”、“垃圾围村”问题。

　　进一步推进节能降耗。加快淘汰落后产能，上半年完成12户重点企业的淘汰任务。实施40个工业节能改造项目，力争节约标准煤15万吨以上。抓紧制定法兰能耗地方标准，促进法兰产业上档升级。实施国家可再生能源建筑应用示范城市项目，完成建筑节能改造25万平方米。打造忻州城市照明节能样板工程，完成3个广场公园、和平街20座楼宇、云中河两岸路灯节能改造任务。

　　进一步强化生态建设。扎实推进中心城市创建国家环保模范城工作，加快原平、神池、五寨、岢岚、河曲、保德等县市创建山西省环保模范城进度。加大造林绿化力度，重点实施“两山”造林工程、“两网”绿化工程和书记、县长精品林工程，完成营造林60万亩以上。高标准完成忻阜高速通道绿化，全面完成大运高速忻州段和五台山核心景区绿化提升，抓好忻保高速、太佳高速绿化。完善九龙岗森林公园建设，提升各县市城郊公园绿化水平。推进干果经济林示范工程，新发展干果经济林10万亩。进一步加大重点流域和矿山生态环境治理修复力度，完成清水河、南云中河生态综合治理提升工程。

　　（五）加快建设文化强市，推动文化大发展大繁荣。

　　贯彻落实文化强市实施意见，推进“十大文化工程”，加快文化资源大市向文化强市迈进。推进社会主义核心价值体系建设，深入开展社会公德、职业道德、家庭美德和个人品德教育，推动学雷锋志愿服务活动常态化，提高全社会文明水平。启动省级文明城镇群创建和学习型城市建设，深入开展“忻州形象”系列征集活动。加快公共文化服务体系建设，完善市、县、乡、村四级文化服务网络。启动忻州市档案馆建设。组织开展“周末大戏台”、“梨花奖舞台艺术大赛”等独创性文化活动，促进文艺创新，推出新人新作，丰富忻州文化品牌。积极组织开展群众性文化活动，认真实施“文化低保工程”和“文化惠民工程”，保障人民群众享受文化成果的基本权益。健全文化遗产保护体系，促进文化遗产的传承、保护和发展。落实文化产业扶持政策，推动文化产业尽快成为我市新的支柱产业。深化文化体制改革，全面整合文化资源，完善国有文化单位转企改制后的体制机制，巩固和扩大改革成果。深入开展“扫黄打非”专项行动，着力繁荣文化市场。

　　（六）切实保障和改善民生，解决关系群众切身利益的问题。

　　进一步稳定和扩大就业。落实更加积极的就业政策，以高校毕业生、农村转移劳动力、城镇就业困难群体为重点，全面做好各类人员的就业工作。加快发展加工制造、服务业等劳动密集型产业，大力发展中小微型企业，多渠道开发就业岗位。鼓励全民创业，以创业带动就业。强化就业培训、困难群体就业援助、就业失业管理等服务工作。坚决查处拖欠和克扣职工工资、养老金等违法行为，维护劳动者合法权益。

　　健全和完善社会保障体系。全面落实社会保障政策，进一步扩大各项社会保险覆盖面，努力提高保障水平。加快推进城镇居民社会养老保险和新农保工作，实现城乡养老保险制度全覆盖。积极稳妥地开展事业单位养老保险制度改革试点。巩固城镇居民医疗保险和新农合参保率，将政府补助标准在去年的基础上每人再提高40元，做好失业人员参加职工医疗保险工作。推进事业单位工作人员参加工伤保险，把老工伤人员纳入工伤保险统筹。高度重视农民工、非公经济组织员工、灵活就业人员参加社会保险工作，从实际出发破解特殊群体参保难题。不断完善被征地农民社会保障政策，将被征地农民纳入相应的社会保险范围。切实关心低收入群体生活，城乡低保对象保障标准每人每月分别提高30元和22元，农村“五保”对象补助标准每人每年提高500元。加快建设社会养老服务体系。积极发展社会福利和慈善事业。

　　加快保障性住房建设。开工建设公共租赁住房、廉租住房、经济适用住房等各类保障性住房19708套，竣工1800套。坚持把工程质量放在首位，切实建成民心工程、德政工程。加快建立保障性住房建设、分配、运营、退出等配套管理制度，确保质量优良、分配公正、管理科学、退出有序。

　　加强和创新社会管理。坚持民生优先、服务为要、基层为重，加强社会管理整体规划设计，抓好社会管理创新项目建设，提高社会管理科学化水平。完善信访工作和矛盾调处机制，畅通群众诉求渠道，切实解决好征地拆迁、企业改制、劳动争议等事关人民群众切身利益的问题，保障人民群众的合法权益，把矛盾纠纷化解在萌芽状态。加强城市社区建设，注重发挥社团组织的作用，进一步夯实社会管理基层基础。强化对流动人口、特殊人群、新经济组织和新社会组织、信息网络等重点领域的管理。健全突发事件应急体系，有效应对各类突发事件。加强防灾减灾工作。加大公路“三超”治理力度。进一步完善社会治安防控体系，深入推进平安创建活动，依法打击各种违法犯罪活动，维护社会和谐稳定。发展老龄事业，做好老干部工作。切实加强人口计生、妇女儿童、人民防空、残疾人和红十字会等工作。

　　（七）大力发展社会事业，促进经济社会协调发展。

　　优先发展教育事业。认真贯彻落实中长期教育改革发展规划纲要，进一步加大教育投入，确保达到财政性教育经费支出占GDP比例4%的规定要求。积极发展学前教育，继续新建、改扩建34所公办标准化幼儿园。进一步加强义务教育学校标准化建设，不断促进义务教育均衡发展，经过3年努力，力争使农村义务教育学校办学水平达到国家和省基本标准，切实提高教学质量。大力发展职业教育，实现中等职业教育免费全覆盖。加快普及高中阶段教育，毛入学率达到88%以上。做好经济困难家庭学生资助工作，真正把资助金发放到困难学生手中。继续实施“一颗鸡蛋学生营养工程”，切实做好五台、繁峙、静乐、神池、五寨、岢岚等6县农村义务教育学校学生营养改善计划试点工作，提高学生营养水平。加强校园安全管理，认真排查学校、幼儿园安全隐患，确保孩子们人身安全。积极发展继续教育、特殊教育，促进各级各类教育全面协调发展。

　　做好科技和人才工作。加大科技投入，完善科技服务体系。鼓励自主创新，全年专利申请量达到620件以上，其中发明专利申请量占到30%以上。大力培育高新技术企业，力争年内达到10户以上。促进市内外产学研的结合，围绕煤炭清洁生产、现代煤化工、新能源、新材料、现代装备制造、绿色农副产品加工等重点产业，开展技术攻关，加快科技成果转化，为产品升级和产业转型提供技术支撑。加大科普教育力度，进一步提高全民科技素质。深入实施人才强市战略，全面落实中长期人才发展规划，大力培养、引进各类实用人才、专业人才、拔尖人才、创新人才和领军人物，统筹推进各类人才队伍建设，为转型跨越发展提供人才保证。

　　加快发展卫生和体育事业。继续深化医药卫生体制改革，不断完善基本医疗保障制度和基本药物制度，加快推进以县级医院为重点的公立医院改革。稳步推进基本公共卫生服务均等化，强化重大疫病预防控制，加大健康教育工作力度，做好妇幼卫生和爱国卫生工作。完善医疗卫生服务体系，加强全科医生培养和乡村卫生人才队伍建设。全面推进医疗卫生基础设施项目建设，市人民医院主体工程要力争完工，市中医院医技康复楼要确保投用，12个县级卫生监督机构建设项目以及河曲、保德、岢岚、繁峙4个县的急救中心建设项目要全面完成。切实加强卫生服务质量管理。全面做好食品药品安全工作。认真组织开展医疗机构对口支援活动。全面贯彻《全民健身实施计划》，抓好全民健身公共服务体系建设。办好第六届“摔跤节”。

　　（八）加大安全生产工作力度，推动安全生产形势持续稳定好转。

　　安全生产责任重于泰山。安全生产只有起点，没有终点。准确把握安全生产的特殊规律，继续坚持行之有效的好经验、好做法，严格按照安全生产规程办事，进一步做好安全生产各项工作，巩固和发展安全生产来之不易的好形势。严格落实企业安全生产主体责任，继续推行企业法人安全生产承诺制。扎实推进企业标准化和信息化建设，不断完善企业安全档案建设，认真实施企业从业人员素质提升工程，努力提高企业本质安全度。严格落实安全生产政府监管责任，实行安全生产挂牌责任制。继续深化安全生产专项整治，深入开展以煤矿、非煤矿山、道路交通和危险化学品等行业为重点，覆盖所有生产领域的整治行动，始终保持严厉打击非法违法生产经营建设行为的高压态势。建立安全隐患举报重奖和安全隐患事故重罚制度，真正把安全隐患置于全社会的监督之下，排除在萌芽状态。加强安全生产监管队伍建设，健全各项规章制度，细化各个环节的监管责任，不断提升安监执法水平。严格安全生产工作考核，进一步加大安全生产事故责任追究力度。加强安全生产宣传教育，深入开展企业安全文化建设和安全知识进家庭活动，在全市上下营造重视安全、做好安全、确保安全的良好社会氛围，形成推动安全生产工作的强大合力。

　　（九）深化改革开放，加快推进转型综改试验区建设。

　　继续深化各项改革。认真做好原平市、保德县扩权强县改革试点工作，下放权力，搞好指导，切实落实县域发展的自主权。深入推进行政审批制度改革，进一步清理和规范行政审批事项，创新行政审批模式，实现一站式审批、一个窗口对外、一条龙服务，切实提高行政效能，创优政务环境。积极推进事业单位改革，全面完成分类工作，继续推进人事管理、收入分配等配套改革，真正激发事业单位活力。稳妥推进企业改革。国有企业改革要从实际出发，在充分尊重企业自主权、坚决维护职工合法权益的基础上，坚持一企一策、分类指导、成熟一个、改制一个的原则，真正建立现代企业制度，切实确立和尊重企业自主经营、自负盈亏的市场主体地位。鼓励引导民营企业加快建立现代企业制度，支持民营企业加强管理团队、营销团队、研发团队建设。深入推进集体林权制度改革，完善公益林补偿等政策。依法规范和促进农村土地承包经营权流转，积极推进适度规模经营。不断优化金融生态环境，严厉打击非法集资活动，切实提高我市存贷比，真正为金融资本支持地方经济发展创造条件。

　　全面推进转型综改试验区建设。加强综改规划指导，制定《忻州市转型综改试验区建设实施方案》。按照政府推动、市场主导、企业主体、社会参与的要求，真正把现有企业打造成现代化企业，以提升促转型；发展现有企业的上下游产品和相关产业，以延伸促转型；引进新企业，建设新项目，以建型促转型，走出具有忻州特色的转型发展路子。大力推进原平市、宁武县和禹王循环经济园区、蓝天科技创新园区以及14个县（市、区）确定的试点企业先行先试工作，支持4大领域22个重大标杆项目的建设工作，全力抓好五台山改造提升工程，力争晋北铝业三期60万吨铝材深加工项目、山西煤运集团宁武年产60万吨烯烃项目等综改重点项目取得新进展。进一步加快综改工作节奏，全面推进发改、经信、科技、人社等11个部门和10大类项目与省里的对接工作，确保我市在全省综改项目大盘子里占有更大份额。积极推进体制机制创新，力争在项目审批、用地保障、资金筹措等方面取得实质性突破，增强转型跨越发展的动力和活力。努力建设“三个门户”，打造“三个集散地”。建设以保德、河曲为重点，面向陕蒙的西部门户；以忻府、定襄、原平为重点，面向太原都市圈的中部门户；以五台、繁峙为重点，面向京冀津的东部门户，充分发挥“三个门户”的桥头堡作用，密切忻州同外部世界的联系，真正把人才、资金、技术、信息引进来，把产品、服务、劳动力输出去，加快形成开门搞综改的生动局面。打造东部的五台山，中部的奇村、顿村、大营、汤头温泉度假区，西部的芦芽山“三个集散地”，加快形成全市一体化大旅游格局。

　　不断扩大对外开放。全面落实“走出年”部署，加大以县为单位的小分队招商引资力度。进一步加强对外交流，切实做好第七届中博会、第四届能博会等重大展会的参展工作，精心组织好第三届中国·五台山国际文化旅游月。加强各县（市、区）之间的合作。依托五保、灵河高速公路和神朔、朔黄铁路，以及各县（市、区）的资源区位优势，发展河曲、保德煤电铝化板块，神池、五寨、岢岚、偏关现代农业板块，宁武、静乐煤电化板块，忻府、定襄、原平多元产业板块，繁峙、代县矿业冶炼板块，五台旅游板块等6大特色区域经济板块，推动板块要素优化整合，提升全市经济的整体竞争力。

　　（十）加强民主法制建设，促进社会公平正义。

　　发展社会主义民主政治。认真执行市人大及其常委会的决议、决定，依法向人大及其常委会报告工作，自觉接受人大监督。积极支持人民政协履行政治协商、民主监督、参政议政职能，广泛听取各民主党派、工商联、无党派人士的意见和建议。高度重视并认真办理人大代表议案、建议和政协委员提案。大力支持工会、共青团、妇联等群团组织开展工作。完善基层民主管理制度，提升基层自治组织的管理能力。深入推进政务公开、村务公开和厂务公开，进一步做好农廉工作，保障人民群众的知情权、参与权、表达权、监督权。认真贯彻落实民族宗教政策。进一步做好外事、侨务、对台工作。支持国防和军队建设，不断引深双拥共建，全面做好优抚安置工作。

　　加强社会主义法制建设。加强和改进政府法制工作，支持人民法院、检察院依法独立公正行使职权，做好法律服务和法律援助工作，维护社会公平正义。加强法制宣传教育，深入推进“六五”普法各项工作，不断增强全民法制意识，真正让守法者不吃亏、违法者受惩处，在全社会形成自觉学法、尊法、守法、用法的良好氛围。

　　深入开展市场秩序整顿。集中整顿土地秩序、建筑秩序、交通秩序、交易秩序，依法严厉打击违法用地、乱搭乱建、违规运输、非法集资等不法行为。加大执法监管力度，重点加强对食品药品价格和医疗、通信、教育等服务收费的监督检查。依法严厉打击制假售假、偷逃骗税、商业欺诈、虚假广告、传销和变相传销等违法活动。深入开展工程建设领域突出问题专项治理，促进工程建设项目高效、安全、廉洁运行。

　　三、切实加强政府自身建设

　　全面做好今年的各项工作，必须进一步加强政府自身建设，不断提高政府的执行力和公信力。

　　树立科学的执政理念。一是树立法治政府的理念。认真贯彻宪法和各项法律法规，坚持在法律和政策的范围内活动。严格按照法定程序行使权力，自觉运用法律政策手段化解社会矛盾，解决实际问题。二是树立为民政府的理念。始终把人民利益放在第一位，深入践行为人民服务宗旨。切实加强公共财政建设，着力抓好煤铁等资源的税、费和基金征收工作，进一步提高土地收益，夯实财政收入基础。积极调整财政支出结构，不断增加民生投入，完善财政资金使用效应评价制度，让人民群众共享改革发展成果。三是树立服务政府的理念。进一步转变政府职能。在全市开展“给人民汇报，向人民问计，请人民评议”活动，真正做到问政于民、问需于民、问计于民，使政府的工作思路、决策部署、方法措施更加符合民意，把政府的作用真正体现在服务企业，服务各团体、各阶层和广大人民群众中。四是树立效能政府的理念。认真落实首问负责制、限时办结制、服务承诺制、责任追究制，改进政府工作作风，以时不我待、只争朝夕的精神做好工作。五是树立诚信政府的理念。加强诚信体系建设，坚决克服各种形式主义，全面兑现各项承诺，切实做到言必行、行必果，用实实在在的工作成效赢得人民群众的信任和支持。六是树立廉洁政府的理念。深化政务公开，进一步完善行政决策制度和程序，自觉接受市委的领导和市人大、市政协、新闻媒体、人民群众的监督，让权力在阳光下运行。弘扬艰苦奋斗优良传统，坚持勤俭办事，反对铺张浪费，继续坚持“三公”支出零增长，进一步降低行政成本。狠抓政府系统廉政建设和反腐败斗争，严肃查处各类违法违纪案件，切实加强政风行风建设。

　　创新政府工作方式。一要坚持依法施政。严格依法行政，坚持运用法律、制度和政策解决问题，推动工作。二要充分发扬民主。认真执行民主集中制原则，努力提高民主决策水平，营造群策群力、精诚团结的工作氛围，依靠集体的力量推动各项工作。三要努力搞好协同。一切在大局下行动，加强各级各部门工作的有效衔接，形成政府工作的整体合力。四要切实严明奖惩。通过精神物质双重激励，进一步调动各级各部门的工作积极性。坚决惩处不作为、乱作为、慢作为、推诿扯皮、执行不力的人和事，切实维护政府权威。五要发挥科技作用。注重运用现代科学技术手段推动工作，提高政府工作的科学化水平。六要真正依靠群众。坚持从群众中来，到群众中去，注重发挥群众的首创精神，不断从人民群众中汲取智慧和力量。

　　各位代表：加强政府自身建设，是做好政府工作的切实保障，是政府工作人员的永恒追求。我们一定要认真落实全市政府系统工作人员“牢记宗旨，甘当公仆；勤奋学习，刻苦工作；恪尽职守，敢讲真话；依法办事，从严治政；严于律己，清正廉洁”的“五章约法”，用实际行动打造“执政为民、敢为人先、群策群力、滴水石穿”的人民信赖的政府，不断提高各级政府的工作水平。

各位代表：今年的政府工作任务繁重，使命光荣，我们一定要在市委的坚强领导下，下定决心，排除万难，全力攻坚，努力完成各项任务，不断开创转型跨越发展新局面，以优异的成绩迎接党的十八大胜利召开！
